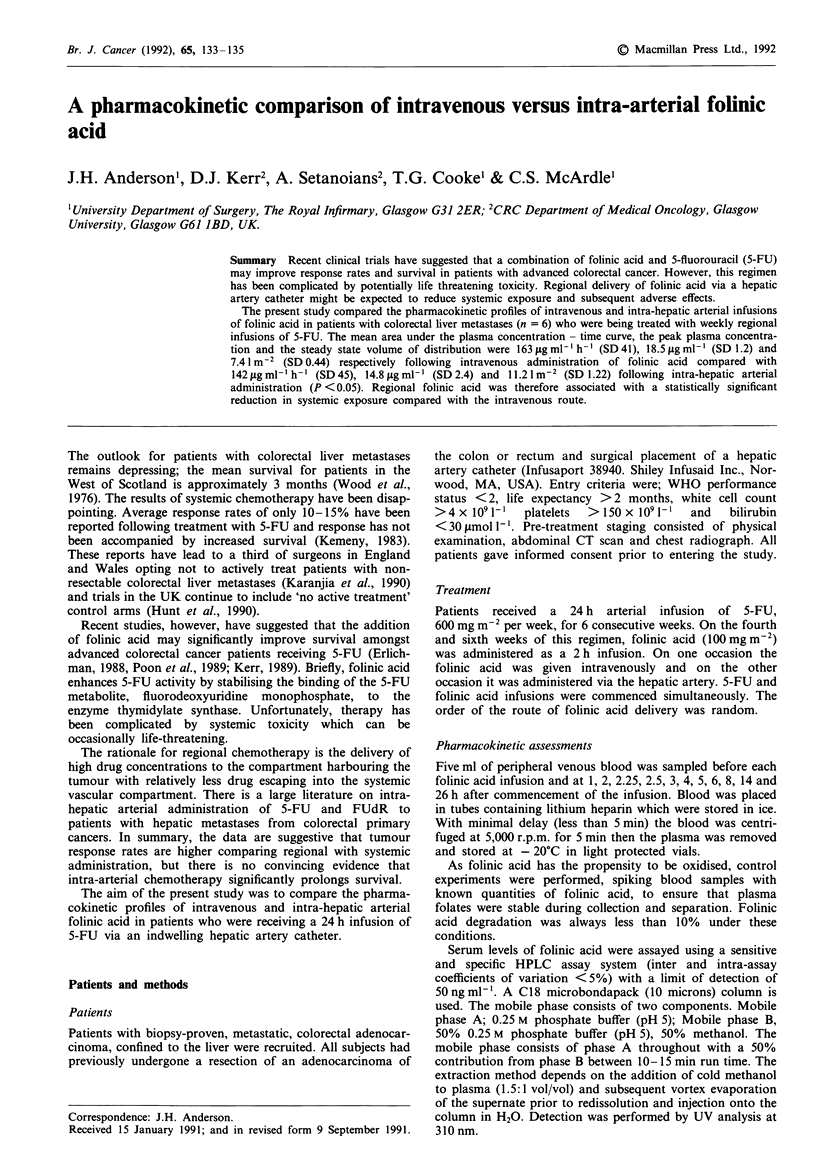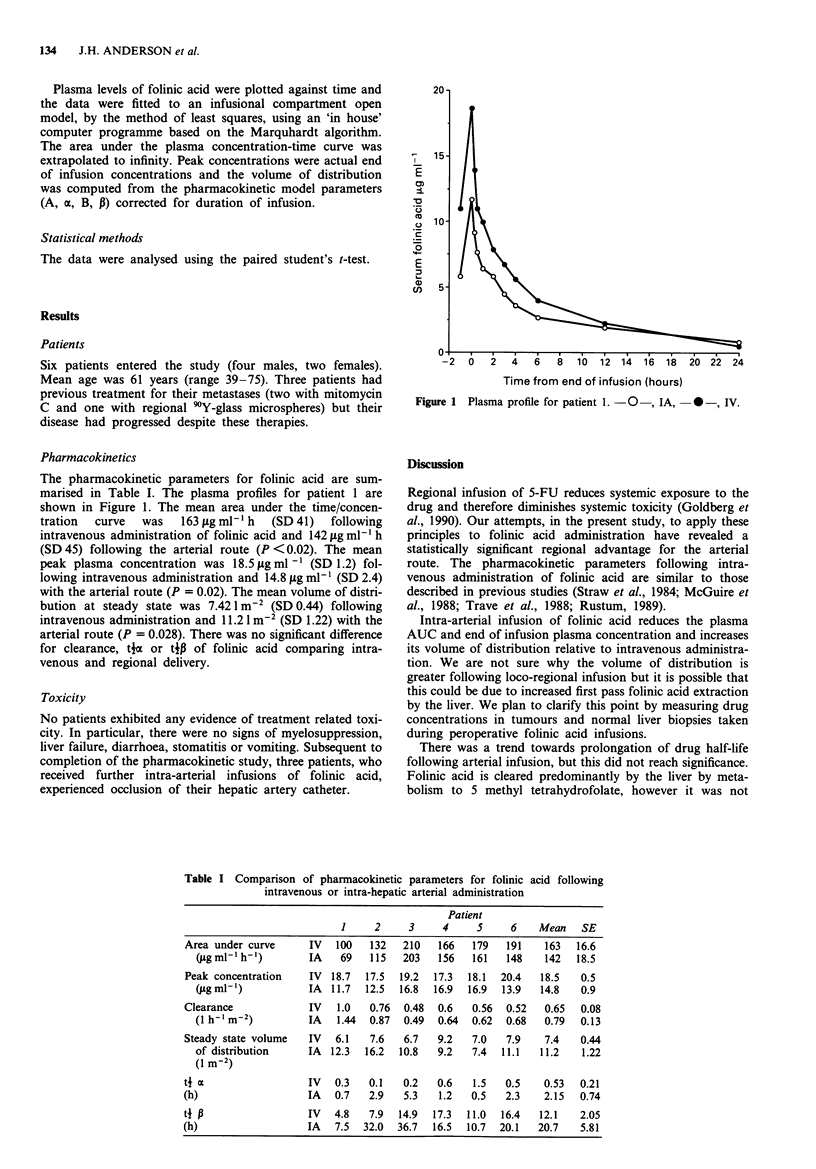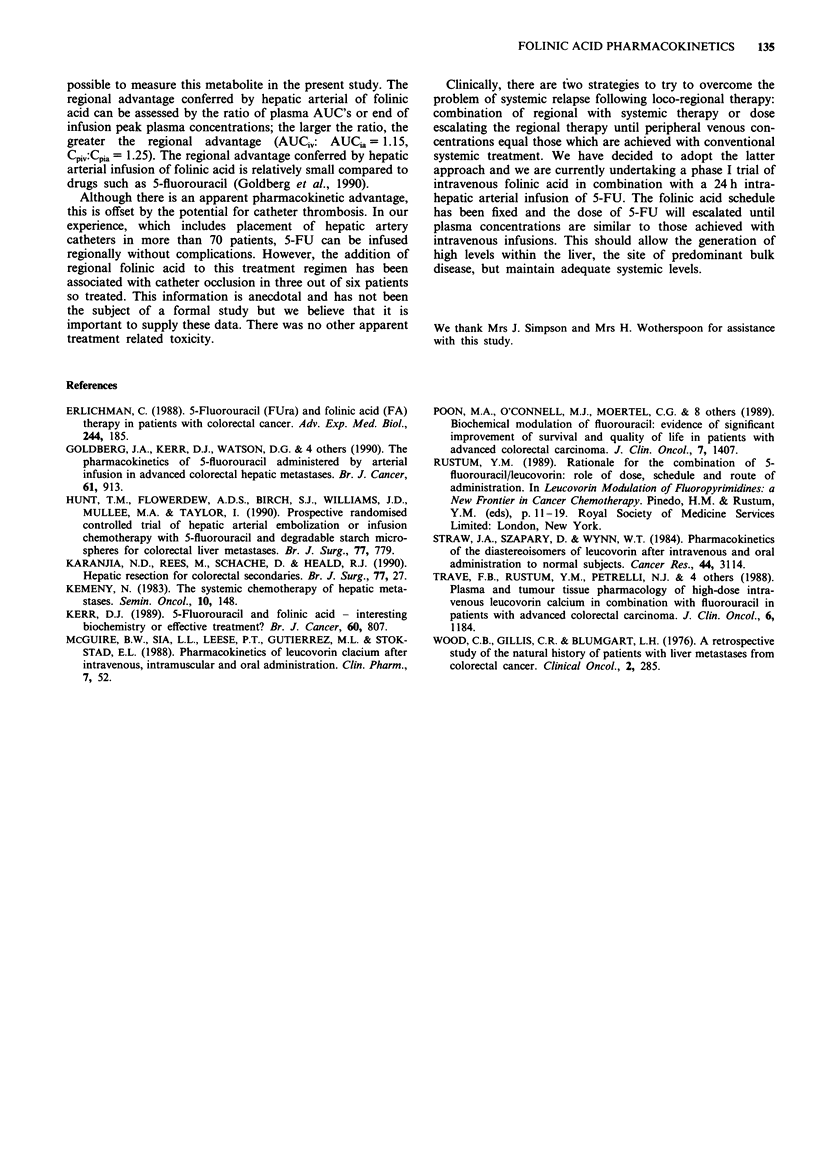# A pharmacokinetic comparison of intravenous versus intra-arterial folinic acid.

**DOI:** 10.1038/bjc.1992.26

**Published:** 1992-01

**Authors:** J. H. Anderson, D. J. Kerr, A. Setanoians, T. G. Cooke, C. S. McArdle

**Affiliations:** University Department of Surgery, Royal Infirmary, Glasgow, UK.

## Abstract

Recent clinical trials have suggested that a combination of folinic acid and 5-fluorouracil (5-FU) may improve response rates and survival in patients with advanced colorectal cancer. However, this regimen has been complicated by potentially life threatening toxicity. Regional delivery of folinic acid via a hepatic artery catheter might be expected to reduce systemic exposure and subsequent adverse effects. The present study compared the pharmacokinetic profiles of intravenous and intra-hepatic arterial infusions of folinic acid in patients with colorectal liver metastases (n = 6) who were being treated with weekly regional infusions of 5-FU. The mean area under the plasma concentration--time curve, the peak plasma concentration and the steady state volume of distribution were 163 micrograms ml-1 h-1 (SD 41), 18.5 micrograms ml-1 (SD 1.2) and 7.41 m-2 (SD 0.44) respectively following intravenous administration of folinic acid compared with 142 micrograms ml-1 h-1 (SD 45), 14.8 micrograms ml-1 (SD 2.4) and 11.21 m-2 (SD 1.22) following intra-hepatic arterial administration (P less than 0.05). Regional folinic acid was therefore associated with a statistically significant reduction in systemic exposure compared with the intravenous route.


Br. J. Cancer (1992), 65, 133–135 © Macmillan Press Ltd., 1992

### A pharmacokinetic comparison of intravenous versus intra-arterial folinic acid

J.H. Anderson[1], D.J. Kerr[2], A. Setanoians[2], T.G. Cooke[1] & C.S. McArdle[1]

[1]*University Department of Surgery, The Royal Infirmary, Glasgow G31 2ER;* [2]*CRC Department of Medical Oncology, Glasgow University, Glasgow G61 1BD, UK.*

**Summary** Recent clinical trials have suggested that a combination of folinic acid and 5-fluorouracil (5-FU) may improve response rates and survival in patients with advanced colorectal cancer. However, this regimen has been complicated by potentially life threatening toxicity. Regional delivery of folinic acid via a hepatic artery catheter might be expected to reduce systemic exposure and subsequent adverse effects.

The present study compared the pharmacokinetic profiles of intravenous and intra-hepatic arterial infusions of folinic acid in patients with colorectal liver metastases ($n$ = 6) who were being treated with weekly regional infusions of 5-FU. The mean area under the plasma concentration – time curve, the peak plasma concentration and the steady state volume of distribution were 163 $\mu g\, ml^{-1}\, h^{-1}$ (SD 41), 18.5 $\mu g\, ml^{-1}$ (SD 1.2) and 7.4 $l\, m^{-2}$ (SD 0.44) respectively following intravenous administration of folinic acid compared with 142 $\mu g\, ml^{-1}\, h^{-1}$ (SD 45), 14.8 $\mu g\, ml^{-1}$ (SD 2.4) and 11.2 $l\, m^{-2}$ (SD 1.22) following intra-hepatic arterial administration ($P < 0.05$). Regional folinic acid was therefore associated with a statistically significant reduction in systemic exposure compared with the intravenous route.

The outlook for patients with colorectal liver metastases remains depressing; the mean survival for patients in the West of Scotland is approximately 3 months (Wood *et al.*, 1976). The results of systemic chemotherapy have been disappointing. Average response rates of only 10–15% have been reported following treatment with 5-FU and response has not been accompanied by increased survival (Kemeny, 1983). These reports have lead to a third of surgeons in England and Wales opting not to actively treat patients with non-resectable colorectal liver metastases (Karanjia *et al.*, 1990) and trials in the UK continue to include 'no active treatment' control arms (Hunt *et al.*, 1990).

Recent studies, however, have suggested that the addition of folinic acid may significantly improve survival amongst advanced colorectal cancer patients receiving 5-FU (Erlichman, 1988, Poon *et al.*, 1989; Kerr, 1989). Briefly, folinic acid enhances 5-FU activity by stabilising the binding of the 5-FU metabolite, fluorodeoxyuridine monophosphate, to the enzyme thymidylate synthase. Unfortunately, therapy has been complicated by systemic toxicity which can be occasionally life-threatening.

The rationale for regional chemotherapy is the delivery of high drug concentrations to the compartment harbouring the tumour with relatively less drug escaping into the systemic vascular compartment. There is a large literature on intra-hepatic arterial administration of 5-FU and FUdR to patients with hepatic metastases from colorectal primary cancers. In summary, the data are suggestive that tumour response rates are higher comparing regional with systemic administration, but there is no convincing evidence that intra-arterial chemotherapy significantly prolongs survival.

The aim of the present study was to compare the pharmacokinetic profiles of intravenous and intra-hepatic arterial folinic acid in patients who were receiving a 24 h infusion of 5-FU via an indwelling hepatic artery catheter.

### Patients and methods

### *Patients*

Patients with biopsy-proven, metastatic, colorectal adenocarcinoma, confined to the liver were recruited. All subjects had previously undergone a resection of an adenocarcinoma of the colon or rectum and surgical placement of a hepatic artery catheter (Infusaport 38940. Shiley Infusaid Inc., Norwood, MA, USA). Entry criteria were; WHO performance status $<2$, life expectancy $>2$ months, white cell count $>4 \times 10^9\, l^{-1}$ platelets $>150 \times 10^9\, l^{-1}$ and bilirubin $<30\, \mu mol\, l^{-1}$. Pre-treatment staging consisted of physical examination, abdominal CT scan and chest radiograph. All patients gave informed consent prior to entering the study.

### *Treatment*

Patients received a 24 h arterial infusion of 5-FU, 600 $mg\, m^{-2}$ per week, for 6 consecutive weeks. On the fourth and sixth weeks of this regimen, folinic acid (100 $mg\, m^{-2}$) was administered as a 2 h infusion. On one occasion the folinic acid was given intravenously and on the other occasion it was administered via the hepatic artery. 5-FU and folinic acid infusions were commenced simultaneously. The order of the route of folinic acid delivery was random.

### *Pharmacokinetic assessments*

Five ml of peripheral venous blood was sampled before each folinic acid infusion and at 1, 2, 2.25, 2.5, 3, 4, 5, 6, 8, 14 and 26 h after commencement of the infusion. Blood was placed in tubes containing lithium heparin which were stored in ice. With minimal delay (less than 5 min) the blood was centrifuged at 5,000 r.p.m. for 5 min then the plasma was removed and stored at $-20°C$ in light protected vials.

As folinic acid has the propensity to be oxidised, control experiments were performed, spiking blood samples with known quantities of folinic acid, to ensure that plasma folates were stable during collection and separation. Folinic acid degradation was always less than 10% under these conditions.

Serum levels of folinic acid were assayed using a sensitive and specific HPLC assay system (inter and intra-assay coefficients of variation $<5\%$) with a limit of detection of 50 $ng\, ml^{-1}$. A C18 microbondapack (10 microns) column is used. The mobile phase consists of two components. Mobile phase A; 0.25 M phosphate buffer (pH 5); Mobile phase B, 50% 0.25 M phosphate buffer (pH 5), 50% methanol. The mobile phase consists of phase A throughout with a 50% contribution from phase B between 10–15 min run time. The extraction method depends on the addition of cold methanol to plasma (1.5:1 vol/vol) and subsequent vortex evaporation of the supernate prior to redissolution and injection onto the column in $H_2O$. Detection was performed by UV analysis at 310 nm.

Correspondence: J.H. Anderson.
Received 15 January 1991; and in revised form 9 September 1991.

Plasma levels of folinic acid were plotted against time and the data were fitted to an infusional compartment open model, by the method of least squares, using an 'in house' computer programme based on the Marquhardt algorithm. The area under the plasma concentration-time curve was extrapolated to infinity. Peak concentrations were actual end of infusion concentrations and the volume of distribution was computed from the pharmacokinetic model parameters (A, $\alpha$, B, $\beta$) corrected for duration of infusion.

### Statistical methods

The data were analysed using the paired student's *t*-test.

### Results

### Patients

Six patients entered the study (four males, two females). Mean age was 61 years (range 39–75). Three patients had previous treatment for their metastases (two with mitomycin C and one with regional $^{90}$Y-glass microspheres) but their disease had progressed despite these therapies.

### Pharmacokinetics

The pharmacokinetic parameters for folinic acid are summarised in Table I. The plasma profiles for patient 1 are shown in Figure 1. The mean area under the time/concentration curve was 163 $\mu$g ml$^{-1}$ h (SD 41) following intravenous administration of folinic acid and 142 $\mu$g ml$^{-1}$ h (SD 45) following the arterial route ($P < 0.02$). The mean peak plasma concentration was 18.5 $\mu$g ml$^{-1}$ (SD 1.2) following intravenous administration and 14.8 $\mu$g ml$^{-1}$ (SD 2.4) with the arterial route ($P = 0.02$). The mean volume of distribution at steady state was 7.42 l m$^{-2}$ (SD 0.44) following intravenous administration and 11.2 l m$^{-2}$ (SD 1.22) with the arterial route ($P = 0.028$). There was no significant difference for clearance, $t\frac{1}{2}\alpha$ or $t\frac{1}{2}\beta$ of folinic acid comparing intravenous and regional delivery.

### Toxicity

No patients exhibited any evidence of treatment related toxicity. In particular, there were no signs of myelosuppression, liver failure, diarrhoea, stomatitis or vomiting. Subsequent to completion of the pharmacokinetic study, three patients, who received further intra-arterial infusions of folinic acid, experienced occlusion of their hepatic artery catheter.

**Figure 1** Plasma profile for patient 1. —○—, IA, —●—, IV.

### Discussion

Regional infusion of 5-FU reduces systemic exposure to the drug and therefore diminishes systemic toxicity (Goldberg *et al.*, 1990). Our attempts, in the present study, to apply these principles to folinic acid administration have revealed a statistically significant regional advantage for the arterial route. The pharmacokinetic parameters following intravenous administration of folinic acid are similar to those described in previous studies (Straw *et al.*, 1984; McGuire *et al.*, 1988; Trave *et al.*, 1988; Rustum, 1989).

Intra-arterial infusion of folinic acid reduces the plasma AUC and end of infusion plasma concentration and increases its volume of distribution relative to intravenous administration. We are not sure why the volume of distribution is greater following loco-regional infusion but it is possible that this could be due to increased first pass folinic acid extraction by the liver. We plan to clarify this point by measuring drug concentrations in tumours and normal liver biopsies taken during peroperative folinic acid infusions.

There was a trend towards prolongation of drug half-life following arterial infusion, but this did not reach significance. Folinic acid is cleared predominantly by the liver by metabolism to 5 methyl tetrahydrofolate, however it was not

**Table I** Comparison of pharmacokinetic parameters for folinic acid following intravenous or intra-hepatic arterial administration

| | | Patient 1 | 2 | 3 | 4 | 5 | 6 | Mean | SE |
|---|---|---|---|---|---|---|---|---|---|
| Area under curve | IV | 100 | 132 | 210 | 166 | 179 | 191 | 163 | 16.6 |
| ($\mu$g ml$^{-1}$ h$^{-1}$) | IA | 69 | 115 | 203 | 156 | 161 | 148 | 142 | 18.5 |
| Peak concentration | IV | 18.7 | 17.5 | 19.2 | 17.3 | 18.1 | 20.4 | 18.5 | 0.5 |
| ($\mu$g ml$^{-1}$) | IA | 11.7 | 12.5 | 16.8 | 16.9 | 16.9 | 13.9 | 14.8 | 0.9 |
| Clearance | IV | 1.0 | 0.76 | 0.48 | 0.6 | 0.56 | 0.52 | 0.65 | 0.08 |
| (l h$^{-1}$ m$^{-2}$) | IA | 1.44 | 0.87 | 0.49 | 0.64 | 0.62 | 0.68 | 0.79 | 0.13 |
| Steady state volume | IV | 6.1 | 7.6 | 6.7 | 9.2 | 7.0 | 7.9 | 7.4 | 0.44 |
| of distribution (l m$^{-2}$) | IA | 12.3 | 16.2 | 10.8 | 9.2 | 7.4 | 11.1 | 11.2 | 1.22 |
| $t\frac{1}{2}\alpha$ | IV | 0.3 | 0.1 | 0.2 | 0.6 | 1.5 | 0.5 | 0.53 | 0.21 |
| (h) | IA | 0.7 | 2.9 | 5.3 | 1.2 | 0.5 | 2.3 | 2.15 | 0.74 |
| $t\frac{1}{2}\beta$ | IV | 4.8 | 7.9 | 14.9 | 17.3 | 11.0 | 16.4 | 12.1 | 2.05 |
| (h) | IA | 7.5 | 32.0 | 36.7 | 16.5 | 10.7 | 20.1 | 20.7 | 5.81 |

possible to measure this metabolite in the present study. The regional advantage conferred by hepatic arterial of folinic acid can be assessed by the ratio of plasma AUC's or end of infusion peak plasma concentrations; the larger the ratio, the greater the regional advantage ($AUC_{iv}$: $AUC_{ia} = 1.15$, $C_{piv}$:$C_{pia} = 1.25$). The regional advantage conferred by hepatic arterial infusion of folinic acid is relatively small compared to drugs such as 5-fluorouracil (Goldberg *et al.*, 1990).

Although there is an apparent pharmacokinetic advantage, this is offset by the potential for catheter thrombosis. In our experience, which includes placement of hepatic artery catheters in more than 70 patients, 5-FU can be infused regionally without complications. However, the addition of regional folinic acid to this treatment regimen has been associated with catheter occlusion in three out of six patients so treated. This information is anecdotal and has not been the subject of a formal study but we believe that it is important to supply these data. There was no other apparent treatment related toxicity.

Clinically, there are two strategies to try to overcome the problem of systemic relapse following loco-regional therapy: combination of regional with systemic therapy or dose escalating the regional therapy until peripheral venous concentrations equal those which are achieved with conventional systemic treatment. We have decided to adopt the latter approach and we are currently undertaking a phase I trial of intravenous folinic acid in combination with a 24 h intrahepatic arterial infusion of 5-FU. The folinic acid schedule has been fixed and the dose of 5-FU will escalated until plasma concentrations are similar to those achieved with intravenous infusions. This should allow the generation of high levels within the liver, the site of predominant bulk disease, but maintain adequate systemic levels.

We thank Mrs J. Simpson and Mrs H. Wotherspoon for assistance with this study.

### References

ERLICHMAN, C. (1988). 5-Fluorouracil (FUra) and folinic acid (FA) therapy in patients with colorectal cancer. *Adv. Exp. Med. Biol.*, **244,** 185.

GOLDBERG, J.A., KERR, D.J., WATSON, D.G. & 4 others (1990). The pharmacokinetics of 5-fluorouracil administered by arterial infusion in advanced colorectal hepatic metastases. *Br. J. Cancer*, **61,** 913.

HUNT, T.M., FLOWERDEW, A.D.S., BIRCH, S.J., WILLIAMS, J.D., MULLEE, M.A. & TAYLOR, I. (1990). Prospective randomised controlled trial of hepatic arterial embolization or infusion chemotherapy with 5-fluorouracil and degradable starch microspheres for colorectal liver metastases. *Br. J. Surg.*, **77,** 779.

KARANJIA, N.D., REES, M., SCHACHE, D. & HEALD, R.J. (1990). Hepatic resection for colorectal secondaries. *Br. J. Surg.*, **77,** 27.

KEMENY, N. (1983). The systemic chemotherapy of hepatic metastases. *Semin. Oncol.*, **10,** 148.

KERR, D.J. (1989). 5-Fluorouracil and folinic acid – interesting biochemistry or effective treatment? *Br. J. Cancer*, **60,** 807.

MCGUIRE, B.W., SIA, L.L., LEESE, P.T., GUTIERREZ, M.L. & STOKSTAD, E.L. (1988). Pharmacokinetics of leucovorin clacium after intravenous, intramuscular and oral administration. *Clin. Pharm.*, **7,** 52.

POON, M.A., O'CONNELL, M.J., MOERTEL, C.G. & 8 others (1989). Biochemical modulation of fluorouracil: evidence of significant improvement of survival and quality of life in patients with advanced colorectal carcinoma. *J. Clin. Oncol.*, **7,** 1407.

RUSTUM, Y.M. (1989). Rationale for the combination of 5-fluorouracil/leucovorin: role of dose, schedule and route of administration. In *Leucovorin Modulation of Fluoropyrimidines: a New Frontier in Cancer Chemotherapy*. Pinedo, H.M. & Rustum, Y.M. (eds), p. 11–19. Royal Society of Medicine Services Limited: London, New York.

STRAW, J.A., SZAPARY, D. & WYNN, W.T. (1984). Pharmacokinetics of the diastereoisomers of leucovorin after intravenous and oral administration to normal subjects. *Cancer Res.*, **44,** 3114.

TRAVE, F.B., RUSTUM, Y.M., PETRELLI, N.J. & 4 others (1988). Plasma and tumour tissue pharmacology of high-dose intravenous leucovorin calcium in combination with fluorouracil in patients with advanced colorectal carcinoma. *J. Clin. Oncol.*, **6,** 1184.

WOOD, C.B., GILLIS, C.R. & BLUMGART, L.H. (1976). A retrospective study of the natural history of patients with liver metastases from colorectal cancer. *Clinical Oncol.*, **2,** 285.